# Positive and negative facial valence perception are modulated differently by eccentricity in the parafovea

**DOI:** 10.1038/s41598-022-24919-7

**Published:** 2022-12-15

**Authors:** Vasilisa Akselevich, Sharon Gilaie-Dotan

**Affiliations:** 1 School of Optometry and Vision Science, Faculty of Life Science, Bar Ilan University, 5290002 Ramat Gan, Israel; 2 The Gonda Multidisciplinary Brain Research Center, Bar Ilan University, Ramat Gan, Israel; 3 UCL Institute of Cognitive Neuroscience, London, UK

**Keywords:** Human behaviour, Emotion, Social behaviour

## Abstract

Understanding whether people around us are in a good, bad or neutral mood can be critical to our behavior, both when looking directly at them or when they are in our peripheral visual field. However, facial expressions of emotions are often investigated at central visual field or at locations right or left of fixation. Here we assumed that perception of facial emotional valence (the emotion’s pleasantness) changes with distance from central visual field (eccentricity) and that different emotions may be influenced differently by eccentricity. Participants (n = 58) judged the valence of emotional faces across the parafovea (≤ 4°, positive (happy), negative (fearful), or neutral)) while their eyes were being tracked. As expected, performance decreased with eccentricity. Positive valence perception was least affected by eccentricity (accuracy reduction of 10–19% at 4°) and negative the most (accuracy reduction of 35–38% at 4°), and this was not a result of speed-accuracy trade-off or response biases. Within-valence (but not across-valence) performance was associated across eccentricities suggesting perception of different valences is supported by different mechanisms. While our results may not generalize to all positive and negative emotions, they indicate that beyond-foveal investigations can reveal additional characteristics of the mechanisms that underlie facial expression processing and perception.

## Introduction

Humans are social creatures. A major part of the information transmitted during human social intercommunication is via non-verbal signals as facial expressions^1^ that convey the physical and emotional states of other people around us. Such facial information may also afford understanding others’ behavior, intentions, and possible reactions and outcomes^2^. While faces are preferably processed when we directly look at them such that they occupy the center of the visual field^3–8^ (but see Refs.^9–12^), in daily life faces do often appear at different locations in the visual field. It is well known that performance of multiple visual functions decreases with eccentricity (the distance from the center of the visual field) and this has been shown for low- to high-level visual functions^13–15^. In a recent study we have found that face discrimination performance that likely taps into face identity perceptual mechanisms, declines with growing eccentricity for neutral faces in the parafovea (≤ 4°)^15^. Multiple studies suggest that the mechanisms supporting the perception of face identity and those supporting the perception of facial expressions may be dissociated^16–20^ such that one neural system in the ventral visual pathway supports face and identity recognition while another neural system located more dorsally (e.g. posterior superior temporal sulcus (pSTS)) supports face and body emotion perception^21–24^. Therefore, even though face discrimination performance declines with growing eccentricity, it is unclear how eccentricity affects perception of facial expressions. While several studies have examined facial expression perception in peripheral vision^25–27^, these studies predominantly examined peripheral locations on the horizontal meridian (right and left of fixation) while information about additional locations across the visual field are still lacking.

Facial expressions of emotions can be classified according to the types of emotions they convey (as anger and happiness)^28^ and also according to their pleasantness (also termed valence)^29^. Pleasant emotions as happiness are classified as having positive valence, unpleasant emotions as fear or disgust are classified as having negative valence, and neutral expression is considered of neutral valence^30^. Here we were interested to examine how facial emotional valence perception is modulated by distance from central (foveal) visual field (≤ 4°) when faces appear in multiple visual field locations not limited to the horizontal meridian. To that end we parametrically tested face valence perception in parafoveal visual field locations (0°, 2°, 4°, as in our earlier study^15^) using faces with positive, neutral, and negative valence while eye movements were being monitored. While peripheral visual performance is known to be reduced due to the cortical magnification factor enhancing central vision representations^31,32^, some studies demonstrate that this could be compensated for by enlargement of peripheral stimuli (e.g. Refs.^8,33^). Since we were interested in examining eccentricity effects on valence perception mimicking as much as possible daily vision conditions, we did not attempt to compensate for parafoveal performance reduction by enlarging parafoveal stimuli. While we expected, in line with other visual functions, that overall face emotional valence perception would decrease with eccentricity, we also assumed that eccentricity may have different effects on different valences if these are supported by dissociated mechanisms, as has been suggested in multiple studies (e.g. Refs.^6,7^).

## Methods

### Participants

58 adults were recruited for this study (54 for the block-design experiment, 38 for the single-trial experiment where 34 of them did both experiments). After excluding two participants from the block-design experiment since they reported difficulties in keeping fixation (see details below), 56 participants (aged 18–37 years (mean age 24.5 $\pm$ 4.75 (SD) years), 33 women, 49 right-handed) with normal or corrected to normal vision (see below) were included in the analysis (51 in the block-design experiment, 37 in the single-trial experiment and 32 of them in both experiments). Of the 54 that were recruited for the block-design experiment, 2 were excluded from the analysis as they reported experiencing difficulties in keeping fixation during the experiment, and additional participant was excluded due technical issues in response acquisition, resulting in 51 participants in the block-design analysis. Of the 38 participants that participated in the single-trial experiment, one was excluded from the analysis since central vision (0°) accuracy in one of the conditions reached floor performance (below chance level) resulting in 37 participants in the single-trial experiment analysis. Of the 34 participants that took part in both experiments, 32 were included in the joint analysis [data of one participant were excluded from the analysis of the single-trial (the one excluded based on floor performance at central vision (0°)) and data of another from the block-design were also excluded (the one excluded due technical issues in response acquisition)]. Visual acuity (VA) measurements (see below) were obtained for all participants but 2 since during the main experimental session the VA testing room was unavailable and these participants did not return for follow-up VA measurements. Cambridge Face Memory Test assessments (see below) were obtained for all participants but 8 since at the time of their testing we did not have access to the online version of the test. Sample size was based on cohort sizes in earlier studies investigating eccentricity effects (Carrasco et al.^13^ (n = 26 in each experiment), Kreichman et al.^15^ (n = 29)) and studies investigating facial expressions in the parafovea with only 14–20 participants (Bayle et al.^34^ with n = 20, Rigoulot et al.^27^ with n = 16, and Smith and Rossit^14^ with n = 14). Sample sizes were set to approximately double these earlier ones (i.e. n = 28–58 per experiment) with a minimum of 30 participants for each within-participant analysis. The experimental protocol was approved by the Bar Ilan University ethics committee, and all the participants signed a written informed consent form in accordance with the Declaration of Helsinki prior to their participation. Participants were reimbursed for their efforts.

### General procedures

The experimental session included visual acuity^35–37^ measurements using a logMAR chart that took a few minutes (for most participants this took place at the beginning of the experimental session, for some between the main experimental sessions). The main experimental part started with a short training session (up to 10 min) with an identical setup and conditions as that of the main experimental task (ending when reaching ≥ 75% accuracy; images different than those used in the main experiment were used in the training). The main experiment followed (in either block- or single-trial design, taking each up to an hour, see details below). Most participants that participated in the block-design study completed it in 2 sessions in 2 different days while most participants in the single-trial study completed it in one session. Participants that took part in both block-design and single-trial studies completed them in 2–3 sessions (most of them undergoing the block-design experiment first), each on a different day. On top of the main experimental sessions participants underwent the Cambridge Face Memory Test (CFMT^38^) for assessing face memory abilities (taking 20–30 min including instructions) using an online version of the test at https://www.testable.org/. Thus, overall, the whole study duration for participants that took part in one of the main experiments (block-design or single-trial) was between 1.5 and 2 h, while for participants that took part in both main experiments it was 2–3 h.

### Emotional face valence experiments

The experiments were created using EyeLink Experiment Builder^®^ software (Mississauga, Ontario, Canada: SR Research Ltd.) running on a Windows 10 operating system, which was used to present stimuli, record and preprocess the data. The stimuli were displayed on an Eizo FG2421 24″ HD (1920 × 1080 pixels, 100 Hz) LCD monitor in a darkened room. After a standard 5-point HV5 calibration, eye movements were monitored throughout the experiment by an EyeLink 1000 DeskTop Mount with binocular recording (only right eye data were used for further analyses) at a sampling rate of 500 Hz. During experimental runs the participant’s head position was stabilized using a chin rest located at a 60 cm viewing distance from the screen. Each face valence experiment began with eye tracker calibration. Using EyeLink Data Viewer (EyeLink Data Viewer 3.2.1 [Computer software]. (2018). Mississauga, Ontario, Canada: SR Research Ltd.) the eye-tracking data, response times, and accuracy were exported to IBM SPSS Statistics27 for Windows (IBM Corp. Released 2020) for further analysis (see below).

### Stimuli

**Figure 1:**
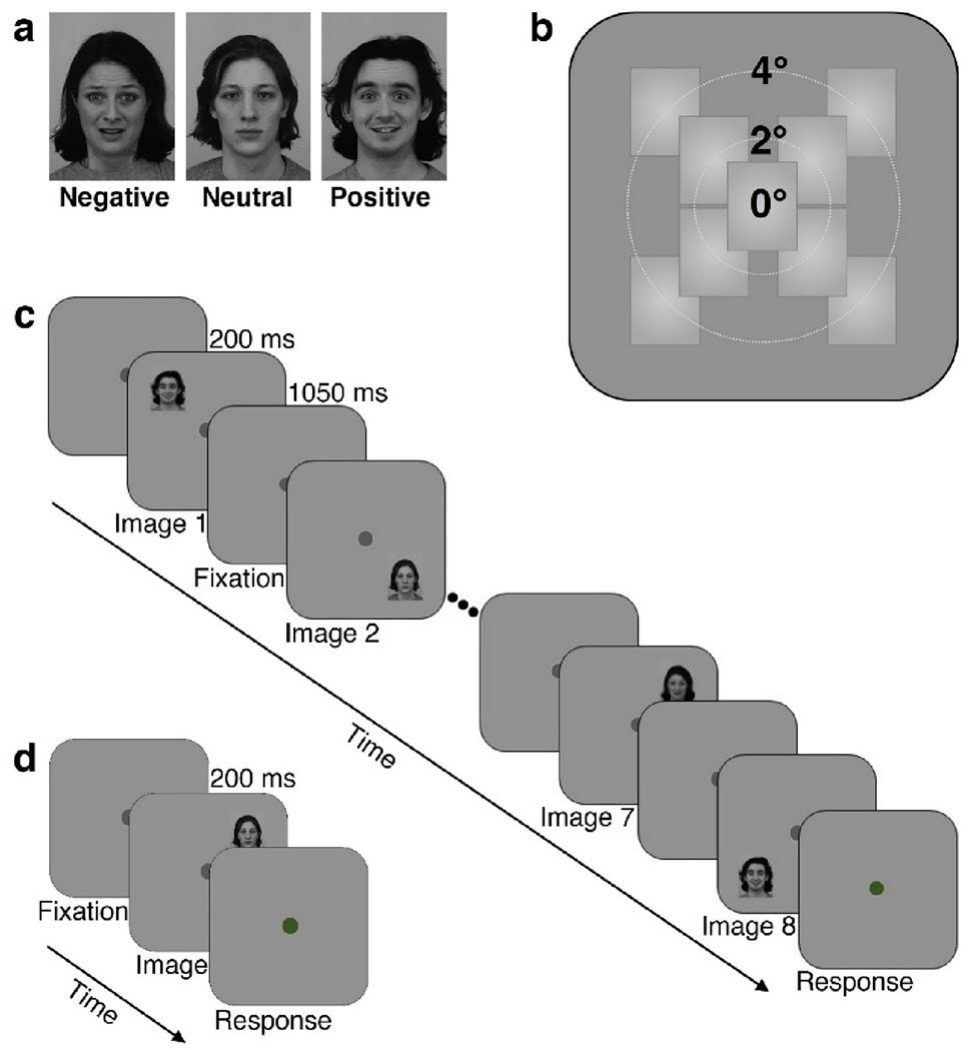
Experimental design. (**a**) Representative stimuli used in the experiments with negative (fearful), neutral, or positive (happy) valence (2° × 2.71°). All stimuli were from the Karolinska Directed Emotional Faces database^39^ (in this figure de-identified image IDs are AF01AFS, AM18NES and AM35HAS). (**b**) 9 possible stimulus locations (central (0°) or parafoveally at 2° or 4° in one of 4 quadrants) appearing against a gray background (each condition was of one main valence appearing at only one eccentricity). (**c**) Block-design experiment: representative positive block at 2° (each block included 8 stimuli (5 with the dominant valence) appearing in pseudo-random order at the block’s eccentricity (2 photos/quadrant in the 2° and 4° blocks). Each block started with a fixation mark at screen center followed by 8 emotional images appearing for 200 ms each with ISI of 1050 ms. The participants were required to fixate and report the dominant valence across the block when the fixation turned green. (**d**) Single-trial experiment: representative neutral trial at 2° where participants initiated each trial (spacebar press) and while keeping fixation had to report the valence of a single stimulus (200 ms) when the green fixation appeared. See “Methods” section for more details.

The images were taken from the Karolinska Directed Emotional Faces database^39^. All 42 stimuli were front view portraits of 14 individuals (7 women and 7 men actors, with the following de-identified database IDs AF01, AF11, AF13, AF17, AF20, AF22, AF24, AM05, AM08, AM10, AM14, AM18, AM25, AM35) looking straight at the camera, each appearing in 3 images depicting either positive (happy/smiling), neutral, or negative (fearful, labeled ‘afraid’ in the Karolinska Directed Emotional Faces database^39^) emotional valence. Since the mouth region may provide distinctive facial expression cues, we chose open mouth photos for the positive and the negative valence conditions. The original color photos were contrast-normalized and converted to grayscale images using the ImageMagick open-source image processing tool (https://imagemagick.org/index.php), similar to earlier studies^40–42^. Image size was calculated based on monitor resolution parameters and viewing distance of 60 cm so that each image would occupy 2° × 2.71° of visual angle (keeping the original proportions of the images: width 562 × height 762 pixels, see Fig. 1a). All peripheral stimuli were presented in the same size and duration as the central stimuli that served as baseline (i.e. optimal performance). Presentation duration was limited for all stimuli to 200 ms to ensure peripheral stimuli were not brought to central vision following an eye movement as was done in our previous study in these locations^15^.

### Block-design experiment

In this experiment we investigated the influence of eccentricity on facial emotional valence perception in a block-design paradigm where each block was of one eccentricity (either 0°, 2°, or 4°, Fig. 1b) and dominated by one valence (positive, neutral, or negative). Each block started with a fixation mark at the screen center for 1050 ms followed by a sequence of 8 emotional images (200 ms/image, 1050 ms ISI, overall 10 s/block, 7 s inter block interval) presented at the block’s eccentricity. 5 of the 8 images in a block displayed the dominant valence (Fig. 1c), two were of another valence and one of the remaining valence, and this was counterbalanced across runs. Valences were balanced per eccentricity. Most participants (40 of the 51) underwent 5–9 blocks per each valence at 0°, and 7–12 blocks per each valence at 2° and at 4° (the rest of the participants completed a smaller number of blocks per condition with a minimum of 2 blocks per each valence at 0°, and 4 blocks per each valence at 2° and at 4° for 6 of the participants). Blocks were clustered into runs, each lasting about 5 min, and participants were allowed to take breaks between runs. At the beginning of each block, a small (0.3° diameter) dark-gray circle that served as a fixation mark was displayed at the center of a gray screen. Participants were instructed to keep fixation across the experiment and refrain from shifting their gaze away from the fixation mark. At the end of each block, the gray fixation mark became green to indicate that a response was expected. Participants were asked to report the most common valence in the block by pressing one of 3 keys on a common keyboard and to guess if unsure (all responses were provided with the right hand regardless of the participant’s hand dominance). Overall, the block-design experimental session took approximately 45 min to 1 h depending on individual between-run breaks and pre-run eye tracking calibration.

### Single-trial experiment

Stimuli were presented with the same size and exposure duration, at the same locations, and with the same emotional valence types as in the block-design experiment. The main difference was that responses were expected after each stimulus presentation to enable evaluating per-face valence perception (Fig. 1b,d). Participants were instructed to maintain fixation on a dark-gray fixation mark in the center of the screen and when feeling ready to press the spacebar to initiate the next trial. Once spacebar was pressed, a stimulus appeared for 200 ms, and the fixation mark turned green to indicate that a response was expected. Participants were asked to report the valence of the presented stimulus as fast and accurate as they could by pressing one of 3 keys and to guess if unsure (all responses were provided with the right hand regardless of the participant’s hand dominance). Once they responded the green circle turned gray again. Each participant underwent 3 runs (each lasting 10–12 min) that each had identical parameters and flow but different stimulus sets (12 unique images in each run: 2 female and 2 male actors, each appearing in 3 emotional expressions). Each of the 12 images appeared twice in each of the 9 locations as depicted in Fig. 1b (one central location (0°), and 8 parafoveal locations (two in each of the four quadrants: at 2° and at 4°)) resulting in 216 trials (12_images_ × 9_locations_ × 2_repetitions/location_) per run, and overall 648 experimental trials with 72 trials/location with 24 valence-specific trials/location. Within run trials were arranged in predefined pseudo-random order, with a restriction that the same location or same valence did not repeat more than twice in a row. Overall, the single-trial experimental session took approximately 40 min to 1 h depending on individual between-run breaks and pre-run eye tracking calibration.

### Analysis

For each experiment mean categorization accuracy and mean response time (regardless of correctness) of every participant were calculated separately for each valence per each eccentricity. In the single-trial experiment, in order to assure that the measured effects represent the perception of the stimuli at the specified visual field locations, per-trial eye movements data were analyzed and only trials in which fixation was kept within 1° distance from screen center were included in the analyses. Specifically, out of 21,996 trials recorded by the eye tracker, in 14,918 trials fixation was kept (i.e. ~ 67.8% of the trials). More specifically, 7078 trials were excluded, and from these 2362 were of negative valence, 2364 of neutral valence, and 2352 of positive valence; 663 at 0°, 2980 at 2°, and 3435 at 4°. Time to respond was not limited and thus no trials were excluded based on response times. For each experiment we ran two separate 2-way repeated-measures ANOVAs (one for accuracy and one for reaction times) with eccentricity (0°, 2°, 4°) and emotional valence (positive, neutral, negative) as factors. Since we found that for each of these analyses outliers (as determined by studentized residual values) did not affect the results (see Supplementary Material for all of these comparisons), we report here results with outliers included (data are also available at the Open Science Framework repository at https://osf.io/8t6r2/). We also performed a 3-way repeated-measures ANOVA on the data of the 32 participants that participated in both experiments to examine the effect of experimental design. Two-tailed correlation analyses were run between individual participant accuracies at 2° (for negative, neutral and positive valences) and individual participant accuracies at 4° (for negative, neutral and positive valences). Statistical analyses were performed with IBM SPSS Statistics27. Greenhouse–Geisser corrections were applied in all cases where Mauchly’s test indicated the assumption of sphericity had been violated. Post-hoc analyses were run with Bonferroni adjustment for multiple comparisons.

## Results

### Block-design experiment

**Figure 2:**
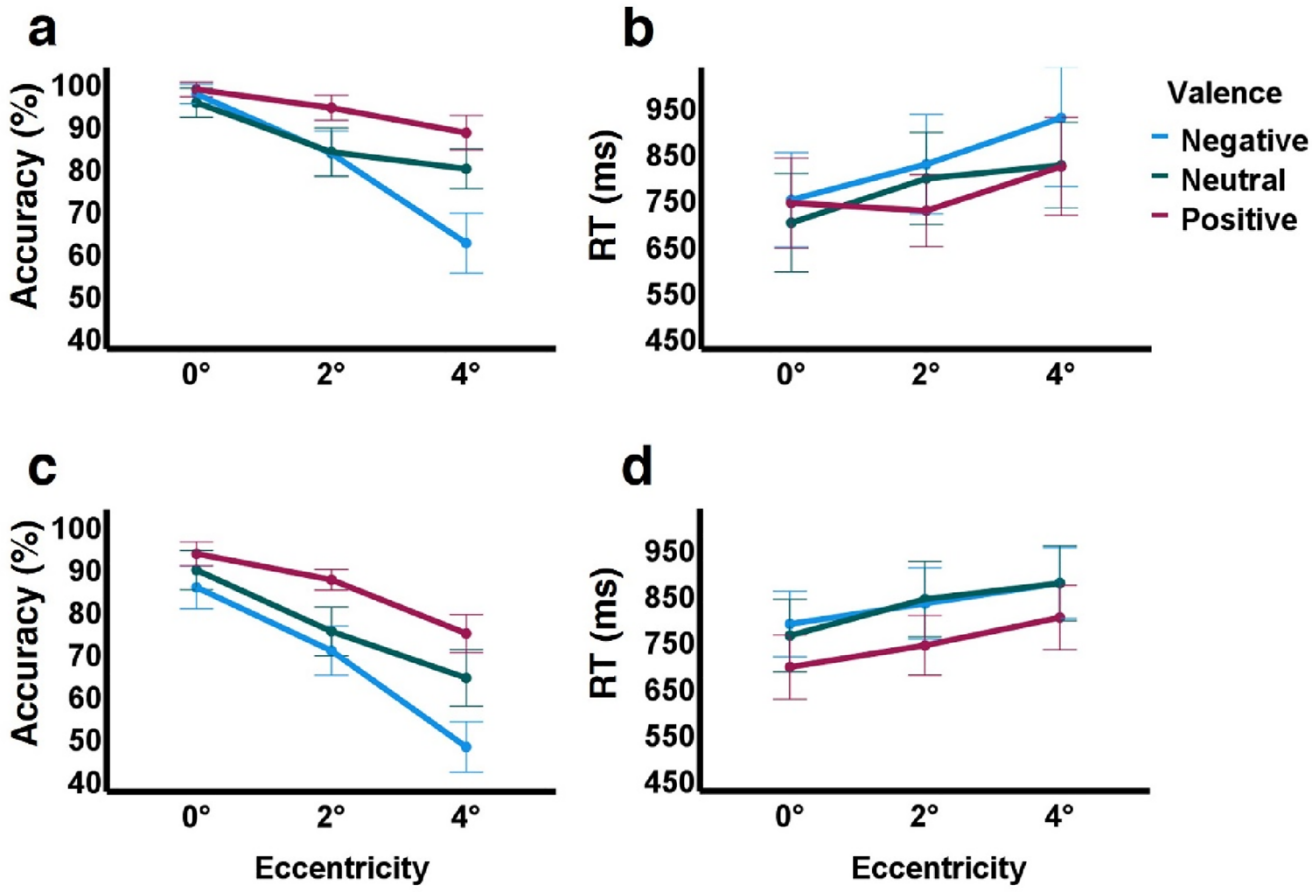
Accuracy and reaction time results for block-design (n = 51) and single-trial (n = 37) experiments. (**a**) Accuracy in block-design experiment (negative valence in blue, neutral in green, positive in red) significantly decreased with eccentricity, with stronger reduction for negative valence (evident by main effect of valence and an interaction between valence and eccentricity). (**b**) Reaction times (RTs) in the block-design experiment were significantly affected by eccentricity but not by valence. (**c**,**d**) Results for single-trial experiment reveal very similar effects on performance to those in the block-design experiment but with poorer average accuracy (see direct comparison in Fig. 3) with negative valence accuracy reaching chance level at 4°. A significant effect of valence on RTs was also observed with faster responses for positive valence. See “Results” section and Tables 1 and 2 for more details.

As we hypothesized, and in line with earlier studies examining eccentricity effects, we found that emotional categorization performance declined with growing eccentricity and this was evident by decreased accuracy (*F*(2, 100) = 94.9, *p* < 0.001, *ŋ*^2^ = 0.655) and slower RTs (*F*(1.642, 82.125) = 10.286, *p* < 0.001, *ε* = 0.821, *ŋ*^2^ = 0.171; see Tables 1, 2, Fig. 2a,b). Specifically we found that accuracy monotonically decreased from central vision (0°) to parafoveal locations of growing eccentricity (accuracy at 0° > accuracy at 2° > accuracy at 4°, all p’s < 0.001). We also found that valence affected accuracy (*F*(2, 100) = 18.29, *p* < 0.001, *ŋ*^2^ = 0.268) such that positive valence accuracy was significantly higher than neutral valence (p < 0.001), and neutral valence accuracy was only marginally higher than that of negative valence (p = 0.061). However, valence effects on accuracy were only evident in the parafovea. For example, responses for positive valence in the parafovea were more accurate than responses for neutral valence (2°: *p* = 0.003, mean difference = 10.4%, 95% CI [3.0, 17.8]; 4°: *p* < 0.005, mean difference = 8.5%, 95% CI [2.1, 14.9]), and neutral and negative valences were similar at 2° (*p* = 1) but at 4° accuracy for neutral valence was significantly higher than that for negative valence (*p* < 0.001, mean difference = 17.5%, 95% CI [7.6, 27.5]). These results were reflected by a significant interaction between eccentricity and emotional valence on accuracy (*F*(3.032, 151.601) = 16.187, *p* < 0.001, *ε* = 0.758, *ŋ*^2^ = 0.245). Interestingly, negative valence categorization accuracy was affected by eccentricity 3-times as much as positive valence accuracy (negative accuracy reduction of 35.1% from 0° to 4° (*p* < 0.001, 95% CI [26.4, 43.8]); positive valence accuracy reduction of 10.2% from 0° to 4° (*p* < 0.001, 95% CI [5.1, 15.3])).

**Table 1:** Results summary for each experiment according to eccentricity and valence.

		Eccentricity	Valence			Mean by eccentricity
			Positive	Neutral	Negative	
Block-design (N = 51)	Accuracy	0°	97.8 ± 6.2	94.7 ± 12.2	96.7 ± 8.2	96.4 ± 5.4
		2°	93.5 ± 10.5	83.1 ± 20.2	82.7 ± 18.8	86.4 ± 11.6
		4°	87.6 ± 14.6	79.1 ± 16.7	61.6 ± 25.1	76.1 ± 12.4
		Mean by valence	93 ± 7.6	85.6 ± 13.3	80.4 ± 14.3	
	RT	0°	735 ± 347	693 ± 379	742 ± 364	724 ± 298
		2°	719 ± 277	789 ± 354	820 ± 383	776 ± 280
		4°	815 ± 377	817 ± 329	920 ± 528	851 ± 355
		Mean by valence	756 ± 279	766 ± 303	827 ± 368	
Single-trial (N = 37)	Accuracy	0°	92.8 ± 8.4	88.9 ± 13.9	84.9 ± 15.1	88.9 ± 8.7
		2°	86.7 ± 7.3	74.5 ± 17.2	69.9 ± 17.3	77 ± 9.2
		4°	74 ± 13.4	63.5 ± 19.98	47.2 ± 17.9	61.6 ± 10.3
		Mean by valence	84.5 ± 7.98	75.6 ± 15.2	67.3 ± 13.4	
	RT	0°	688 ± 209	756 ± 235	781 ± 212	741 ± 197
		2°	734 ± 194	835 ± 245	825 ± 231	798 ± 213
		4°	795 ± 209	869 ± 243	869 ± 230	844 ± 216
		Mean by valence	738 ± 190	819 ± 225	825 ± 193	

Accuracy in % correct, RTs in ms. Different valence conditions in different columns, eccentricity presented in different rows. Values are mean ± SD.

**Table 2:** Statistical analyses summary per experiment.

Measure		Factor		Conditions compared	Mean difference [95% conf. limits]	Sig. (Bonf.)
Block-design (N = 51)	Accuracy	Eccentricity	*F(*2, 100) = 94.9, ***p* < 0.001**, *ŋ*^2^ = 0.655			
				0°, 2°	9.98% [6.9, 13.1]	***p* < 0.001**
				2°, 4°	10.3% [6.4, 14.3]	***p* < 0.001**
		Valence	*F*(2, 100) = 18.29, ***p* < 0.001**, *ŋ*^2^ = 0.268			
				Positive, neutral	7.3% [2.7, 11.95]	***p* < 0.001**
				Neutral, negative	5.28% [−0.18, 10.7]	*p* = 0.061
		Interaction	*F*(3.032, 151.601) = 16.187, ***p* < 0.001**, *ε* = 0.758, *ŋ*^2^ = 0.245			
			Positive	0°, 2°	4.3% [0.73, 7.9]	***p* = 0.013**
				2°, 4°	5.9% [0.41, 11.35]	***p* = 0.031**
			Neutral	0°, 2°	11.6% [6, 17.2]	***p* < 0.001**
				2°, 4°	3.95% [−2.6, 10.5]	*p* = 0.429
			Negative	0°, 2°	14% [8.3, 19.7]	***p* < 0.001**
				2°, 4°	21.1% [14, 28.2]	***p* < 0.001**
	RT	Eccentricity	*F*(1.642, 82.125) = 10.286, ***p* < 0.001**, *ε* = 0.821, *ŋ*^2^ = 0.171			
				0°, 2°	− 52 ms [−127, 23]	*p* = 0.274
				2°, 4°	− 75 ms [−126, − 24]	***p* = 0.002**
		Valence	*F*(1.77, 88.62) = 2.845, *p* = 0.07*, ε* = 0.889, *ŋ*^2^ = 0.054			
		Interaction	*F*(3.237, 161.843) = 1.101, *p* = 0.353, *ε* = 0.809, *ŋ*^2^ = 0.022			
Single-trial (N = 37)	Accuracy	Eccentricity	*F*(1.453, 52.298) = 208.7, ***p* < 0.001**, *ε* = 0.726, *ŋ*^2^ = 0.853			
				0°, 2°	11.9% [8.6, 15,2]	***p* < 0.001**
				2°, 4°	15.4% [13.1, 17.8]	***p* < 0.001**
		Valence	*F*(2, 72) = 19.795, ***p* < 0.001**, *ŋ*^2^ = 0.355			
				Positive, neutral	8.8% [2.4, 15.3]	***p* = 0.005**
				Neutral, negative	8.3% [0.261, 16.3]	***p* = 0.041**
		Interaction	*F*(2.754, 98.824) = 9.082, ***p* < 0.001**, *ε* = 0.686, *ŋ*^2^ = 0.201			
			Positive	0°, 2°	6.1% [2.5, 9.8]	***p* < 0.001**
				2°, 4°	12.7% [8.5, 16.9]	***p* < 0.001**
			Neutral	0°, 2°	14.5% [9.6, 19.4]	***p* < 0.001**
				2°, 4°	10.98% [5.8, 16.2]	***p* < 0.001**
			Negative	0°, 2°	14.97% [7.5, 22.4]	***p* < 0.001**
				2°, 4°	22.7% [17.7, 27.7]	***p* < 0.001**
	RT	Eccentricity	*F*(1.415, 50.95) = 13.666, ***p* < 0.001**, *ε* = 0.708, *ŋ*^2^ = 0.275			
				0°, 2°	− 57 ms [−95, −18]	***p* = 0.002**
				2°, 4°	− 46 ms [−90, −3]	***p* = 0.033**
		Valence	*F*(2, 72) = 21.519, ***p* < 0.001**, *ŋ*^2^ = 0.374			
				Positive, neutral	− 81 ms [−120, −42]	***p* < 0.001**
				Neutral, negative	− 5 ms [−42, 32]	*p* = 1
		Interaction	*F*(2.587, 93.119) = 0.505, *p* = 0.652, *ε* = 0.647, *ŋ*^2^ = 0.014			

For each experiment results of two 2-way repeated-measures ANOVAs (3 × 3) measuring the effects of eccentricity (0°, 2°, 4°) and valence (positive/neutral/negative) on accuracy or RTs (post-hoc analyses based on Bonferroni corrections). Mean difference and 95% confidence interval [lower bound, upper bound] in Mean difference column. Significant results in bold. As can be seen significant effects of eccentricity and valence on accuracy were found in both experiments as well as a significant interaction between them). Eccentricity significantly affected RT in both experiments, but valence only in the single-trial experiment with faster responses for positive valence. See also Fig. 2.

Slower RTs were evident only within the parafovea. Specifically, RTs at 2° were not significantly longer compared to those at 0° (*p* = 0.274), but RTs at 4° were significantly longer compared to 2° (*p* = 0.002, mean difference = 75 ms, 95% CI [24, 126]). No effect of valence on RT was found (*F*(1.77, 88.62) = 2.845, *p* = 0.07, *ε* = 0.889, *ŋ*^2^ = 0.054) and there was no significant interaction between eccentricity and valence on RT (*F(*3.237, 161.843) = 1.101, *p* = 0.357, *ε* = 0.809, *ŋ*^2^ = 0.022; more details in Tables 1, 2).

### Single-trial experiment

Since central vision accuracy (i.e. at fixation (0°)) in the block-design experiment was almost at ceiling (see Fig. 2a), we designed a single-trial experiment. The single-trial experiment aimed to reveal further differences in the effects of eccentricity on valence perception (even at central vision) and potential elevated performance in the parafovea (e.g. possibly resulting from spatio-temporal summation). Here we assumed that performance may decrease (lower accuracies and slower RTs) relative to performance in the block-design experiment.

As expected and can be seen in Fig. 2c, eccentricity effects on accuracy in the single-trial experiment were consistent with the block-design results (compare Fig. 2a,c), and this was evident by monotonic reduction in accuracy as eccentricity grew (*F*(1.453, 52.298) = 208.7, *p* < 0.001, *ε* = 0.726, *ŋ*^2^ = 0.853; see Table 2, Fig. 2 for more details) and slower responses (*F*(1.415, 50.95) = 13.666, *p* < 0.001, *ε* = 0.708, *ŋ*^2^ = 0.275; Tables 1, 2, Fig. 2c,d). Valence effects on accuracy in the single-trial experiment were also similar to those observed in the block-design experiment (compare Fig. 2c to a). Positive valence responses were more accurate than those for neutral valence trials (at 0°: *p* = 0.36; at 2°: *p* < 0.001, mean difference = 12.2%, 95% CI [4.9, 19.5]; at 4°: *p* = 0.02, mean difference = 10.5%, 95% CI [1.3, 19.7]), as in the block-design experiment. Mean accuracy for neutral and negative valences were similar at 2° (*p* = 0.8) but at 4° accuracy of neutral valence was higher than that of negative valence (*p* = 0.003, mean difference = 16.3%, 95% CI [5 to 27.6]). In line with the block-design results here we also found a significant two-way interaction between eccentricity and valence on accuracy (*F*(2.754, 98.824) = 9.082, *p* < 0.001, *ε* = 0.686, *ŋ*^2^ = 0.201), and this was evident by a twofold greater reduction in accuracy for the negative valence from 0° to 4° relative to that of positive valence accuracy (negative valence reduction from 0° to 4° by 37.7% (*p* < 0.001, 95% CI [28.8, 46.5]); positive valence reduction from 0° to 4° by 18.8% (*p* < 0.001*,* 95% CI [13.5, 24.1])).

As in the block-design experiment, here we found that eccentricity significantly affected RT (*F*(1.415, 50.95) = 13.666, *p* < 0.001, *ε* = 0.708, *ŋ*^2^ = 0.275) and this was evident by monotonic increase of RTs with growing eccentricity (RTs at 2° significantly longer than at 0° (*p* = 0.002, mean difference = 57 ms, 95% CI [18, 95]), RTs at 4° significantly longer than at 2° (*p* = 0.033, mean difference = 46 ms, 95% CI [3, 90])). Furthermore, while in the block-design experiment valence only marginally affected RTs (p = 0.07), here we found a significant effect of valence on RTs (*F*(2, 72) = 21.519, *p* < 0.001, *ŋ*^2^ = 0.374) which was evident by faster responses to positive valence relative to neutral valence (*p* < 0.001, mean difference =  − 81 ms, 95% CI [− 120, − 42]), while neutral valence and negative valences RTs were similar (*p* = 1). No significant interaction between eccentricity and valence were found on RTs (*F*(2.587, 93.119) = 0.505, *p* = 0.652, *ε* = 0.647, *ŋ*^2^ = 0.014; see also Table 2).

### Comparing block-design and single-trial results

**Figure 3:**
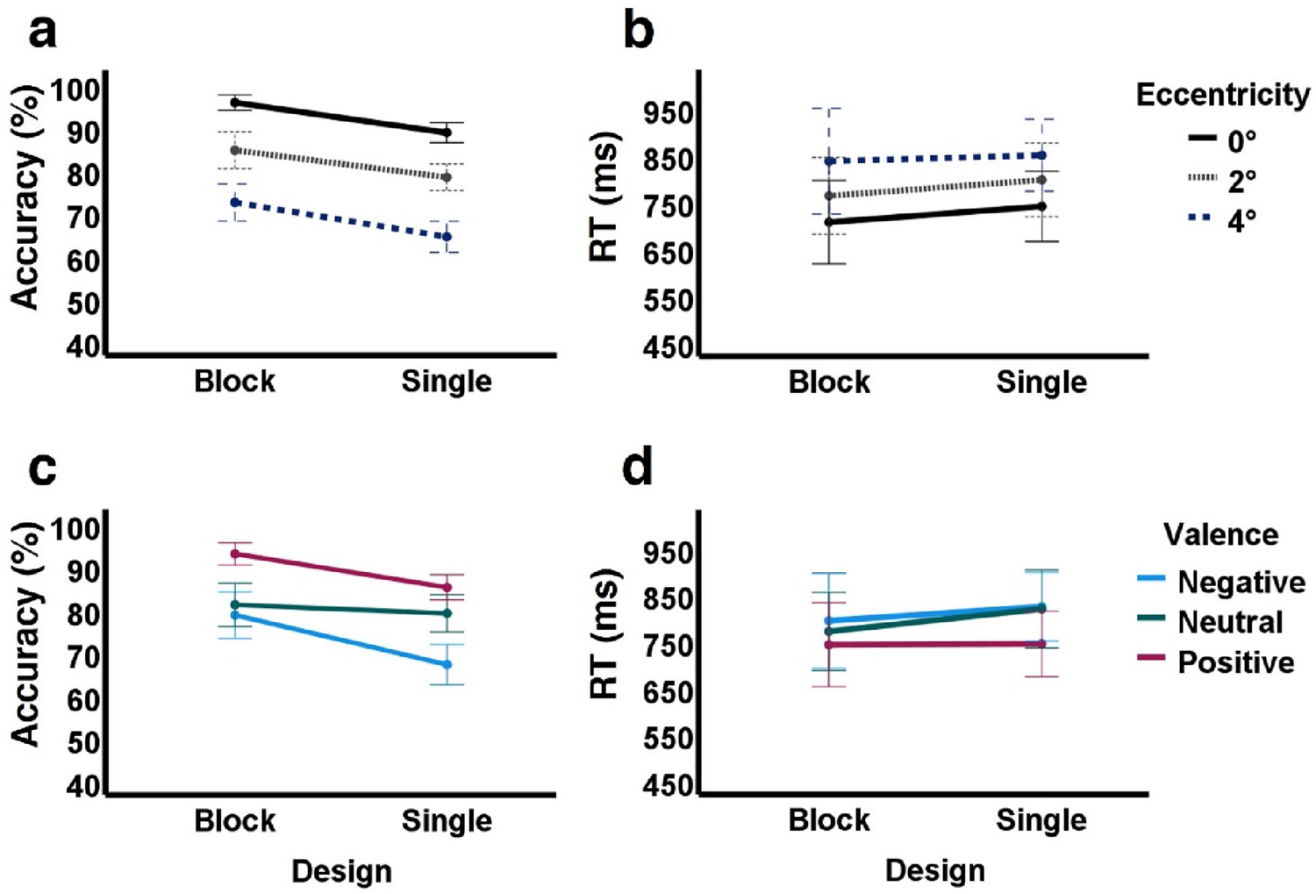
Block-design vs single-trial within-participant accuracy and reaction time comparisons (n = 32). (**a**,**b**) Design by eccentricity interaction plots for accuracy (**a**) and RTs (**b**); (**c**,**d**) design by valence interaction plot for accuracy (**c**) and RTs (**d**). These direct between-experiment comparisons revealed single-trial accuracy was significantly lower than that of the block-design experiment (possibly reflecting spatiotemporal summation) but no RT differences were found. See “Results” section and Table 3 for more details.

Since performance for the single-trial experiment was on average poorer than that of the block-design experiment (overall lower accuracy and longer RTs), we directly compared the results of these experiments. We hypothesized that these differences could result from spatio-temporal summation (information buildup) during block presentation. A 3-way repeated-measures ANOVA with experimental design (block/single-trial), eccentricity, and valence as factors on accuracy of the 32 participants that took part in both experiments confirmed that there was a significant effect of experimental design (*F*(1, 31) = 28.069, *p* < 0.001, *ŋ*^2^ = 0.475) with lower accuracy in the single-trial (*p* < 0.001, mean difference =  − 7.13%, 95% CI [− 9.9, − 4.4]) as can be seen in Fig. 3, panels a and c. In line with our results in both the block-design and the single-trial experiments, here too we found significant main effects of eccentricity (*F*(2, 62) = 195.1, *p* < 0.001, *ŋ*^2^ = 0.863, see Fig. 3a) and emotional valence (*F*(2, 62) = 22.593*, p* < 0.001, *ŋ*^2^ = 0.422, see Fig. 3c) as well as 2-way interaction between valence and eccentricity (*F*(2.81, 87.21) = 17.71, *p* < 0.001, *ε* = 0.703, *ŋ*^2^ = 0.364); see Table 3. Furthermore, no 3-way interaction (*F*(2.815, 87.27) = 0.585, *p* = 0.616, *ε* = 0.704, *ŋ*^2^ = 0.019) or 2-way interaction between design and eccentricity (*F*(2, 62) = 0.47, *p* = 0.627, *ŋ*^2^ = 0.015) were found (see Fig. 3, Table 3 for more details). Note that the significant 2-way interaction between experimental design and valence (*F*(2, 62) = 7.64, *p* = 0.001, *ŋ*^2^ = 0.198) reflects a different change in accuracy for the different valences: while positive and negative valences had lower accuracy in single-trial (*p* < 0.001), neutral valence accuracy was not affected by experimental design (*p* = 0.267).

**Table 3:** Block-design vs single-trial within-subject statistical comparisons (n = 32).

	Factor		Conditions compared	Mean difference [95% conf. lim.]	Sig. (Bonf.)
Accuracy	Design	*F*(1, 31) = 28.069, ***p* < 0.001**, *ŋ*^2^ = 0.475			
			Block, single	7.13% [4.4, 9.9]	***p* < 0.001**
	Eccentricity	*F*(2, 62) = 195.1, ***p* < 0.001**, *ŋ*^2^ = 0.863			
			0°, 2°	10.78% [8, 13.5]	***p* < 0.001**
			2°, 4°	13% [10.34, 15.77]	***p* < 0.001**
	Valence	*F*(2, 62) = 22.593, ***p* < 0.001**, *ŋ*^2^ = 0.422			
			Positive, neutral	8.98% [3.7, 14.25]	***p* < 0.001**
			Neutral, negative	7.18% [0.35, 14]	***p* = 0.037**
	**3-way interaction**				
	Design × eccentricity × valence	*F*(2.815, 87.27) = 0.585, *p* = 0.616, *ε* = 0.704, *ŋ*^2^ = 0.019			
	**2-way interactions**				
	Design × eccentricity	*F(*2, 62) = 0.47, *p* = 0.627, *ŋ*^2^ = 0.015			
	Design × valence	*F*(2, 62) = 7.64, ***p* = 0.001**, *ŋ*^2^ = 0.198			
		Positive	Block, single	7.82% [4.5, 11.15]	***p* < 0.001**
		Neutral	Block, single	2% [−1.6, 5.62]	*p* = 0.267
		Negative	Block, single	11.6% [6.7, 16.41]	***p* < 0.001**
	Eccentricity × valence	*F*(2.81, 87.21) = 17.71, ***p* < 0.001**, *ε* = 0.703, *ŋ*^2^ = 0.364			
RT	Design	*F*(1, 31) = 0.396, *p* = 0.534, *ŋ*^2^ = 0.013			
	Eccentricity	*F*(2, 62) = 16.663, ***p* < 0.001**, *ŋ*^2^ = 0.35			
			0°, 2°	− 56 ms [−111, −2]	***p* < 0.001**
			2°, 4°	− 63 ms [−104, −22]	***p* = 0.001**
	Valence	*F*(2, 62) = 8.558, ***p* = 0.001**, *ŋ*^*2*^ = 0.216			
			Positive, neutral	− 52 ms [−87, −166]	***p* = 0.002**
			Neutral, negative	− 14 ms [−54, 26]	*p* = 1
	**3-way interaction**				
	Design × eccentricity × valence	*F*(3.201, 99.234) = 1.248, *p* = 0.297, *ε* = 0.8, *ŋ*^2^ = 0.039			
	**2-way interactions**				
	Design × eccentricity	*F*(1.543, 47.834) = 0.189, *p* = 0.771, *ε* = 0.772, *ŋ*^2^ = 0.006			
	Design × valence	*F*(2, 62) = 1.002, *p* = 0.373, *ŋ*^2^ = 0.031			
	Eccentricity × valence	*F*(2.572, 79.734) = 1.171, *p* = 0.323, *ε* = 0.643, *ŋ*^2^ = 0.036			

Results of two 2 × 3 × 3 3-way repeated-measures ANOVAs (one for accuracy, one for RTs), each with experimental design (block/single-trial), eccentricity (0°, 2°, 4°), and valence (positive/neutral/negative) as within-subject factors (n = 32 that participated in both experiments). Post-hoc analyses with Bonferroni corrections, significant results in bold, conventions as in Table 2. See also Fig. 3. As can be seen, the results replicate those found in each experiment for both accuracy and RTs, and an effect of experimental design was only found for accuracy due to lower accuracy in the single-trial experiment. Significant 2-way interaction of eccentricity and valence on accuracy reflects effects already reported for each experiment separately (e.g. see Table 2).

For RTs, the only significant effects were of eccentricity and valence (*F*(2, 62) = 16.663*, p* < 0.001, *ŋ*^2^ = 0.35*; F*(2, 62) = 8.558*, p* = 0.001*, ŋ*^2^ = *0.2*16, see Fig. 3 panels b,d) in line with the previous experimental findings, while experimental design did not affect RTs (*F*(1, 31) = 0.396*, p* = 0.534*, ŋ*^2^ = 0.013). No 3-way interaction on RT (*F*(3.201, 99.23) = 1.248, *p* = 0.297, *ε* = 0.8, *ŋ*^2^ = 0.039) or 2-way interactions (design × eccentricity: *F*(1.543, 47.834) = 0.189, *p* = 0.771, *ε* = 0.772, *ŋ*^2^ = 0.006; design × valence: *F*(2, 62) = 1.002, *p* = 0.373, *ŋ*^2^ = 0.031; eccentricity × valence: *F*(2.572, 79.734) = 1.171, *p* = 0.323, *ε* = 0.643, *ŋ*^2^ = 0.036) were found (see Table 3 for more details).

### Cross-conditions analyses

**Figure 4:**
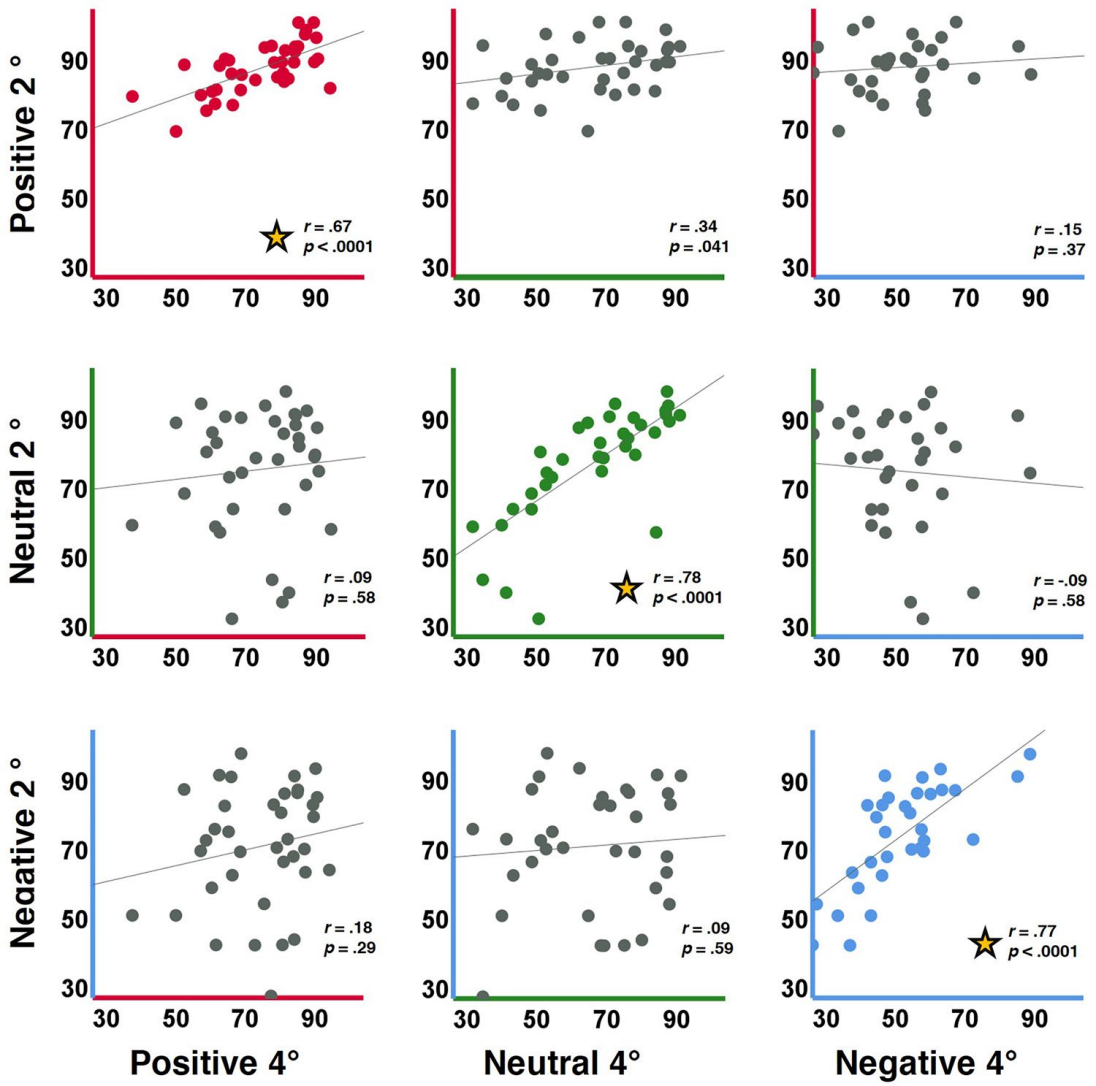
Within-valence but not between-valences performance is correlated across eccentricities (n = 37). Each scatterplot represents a comparison between parafoveal accuracies (2° on the y-axis by valence, 4° on the x-axis by valence). In each scatterplot each point represents performance of one participant in the single-trial experiment. R values represent correlation values, and p indicates non-corrected correlation significance (Bonferroni corrected p = 0.0056). Note that only within-emotion correlations (presented on the diagonal) were significant (surviving multiple comparisons correction, in bold, denoted by asterisks).

We reasoned that if the mechanisms supporting emotional valence categorization are valence-specific, then within-valence performance should be correlated across eccentricities, but not across valences. Therefore, accuracy performance of 37 participants in each valence condition at eccentricity 2° was compared via correlation analyses to performance in each valence at eccentricity 4° (Fig. 4). These across-eccentricity analyses revealed significant correlations within emotional valence (2° to 4° for positive *r*(35) = 0.67, *p* < 0.0001, for neutral *r*(35) = 0.78, *p* < 0.0001, and for negative *r*(35) = 0.77, *p* < 0.0001, all these surviving multiple comparisons correction) but not between-emotional valence (2° for one emotional valence with 4° for another emotional valence, all *p*’s > 0.29 but *p* = 0.04 for positive 2° to neutral 4°, none of these survived multiple comparisons (n = 9) correction). Interestingly, for RTs we found that all across-eccentricity correlations came out as significant when all responses (regardless of correctness) were included (all *r*’s ≥ 0.65, all *p*’s < 0.001) and also when only correct responses were included (all *r*’s ≥ 0.55, all *p*’s < 0.001). This may suggest that RTs may reflect individual response speed tendency.

### Controlling for potential confounding factors

Since we found that the lowest accuracy was for negative valence and the highest for positive valence, we wanted to examine if these reflected genuine valence judgements or possibly response biases towards positive valence or away from negative valence. To examine these possibilities, we analyzed the error distribution of the single-trial results. We reasoned that if a response bias towards “positive valence” existed then it should be reflected by both more accurate responses when positive valence was presented (higher proportion of “hits” for positive valence) and also in more erroneous “positive” responses when a non-positive valence was presented (i.e. more “false alarms” of positive valence when the valence was not positive). Following the same logic, if a response bias avoiding “negative valence” existed it should be evident both in less accurate responses when negative valence was presented (decreased proportion of hits) and in less erroneous “negative” responses (decreased proportion of false alarms of “negative” when the valence was not negative). As can be seen in Table 4, in all conditions and all eccentricities there were fewer errors made towards “positive” valence (indicating that it was not a prevailing tendency to respond “positive”), and in half of the conditions most errors were made towards “negative” valence (indicating that participants did not refrain from answering “negative”). If our results were affected by response biases (towards positive or refraining from negative responses), such effects would be most evident during the neutral condition. Therefore, we further specifically compared the frequencies of the errors made during the neutral valence condition. Using the chi-square goodness of fit test we examined if error frequencies were statistically different between positive response options and negative response options in the neutral condition. In this analysis we found that errors were not equally distributed and importantly that more negative response choices (responses, i.e. higher frequency) were made relative to positive response choices (*χ*^2^ (1) = 183.68, *p* < 10^–10^). We also examined using the chi-square goodness of fit test whether there were any differences between valences in the frequencies of excluded trials (based on fixation criteria) and found no significant differences between valences (for all eccentricities (*χ*^2^(2) = 0.12, *p* = 0.94)). These results indicate that our findings of higher performance for positive valence are not a consequence of specific response biases and genuinely reflect valence judgements.

**Table 4:** Wrong responses distribution by eccentricity and valence.

Conditions			Wrong responses			Sum of errors 4053
Eccentricity	Valence of displayed stimuli	Total trials 14,918	Positive	Neutral	Negative	
0°	Positive	591	–	**20**	16	36
	Neutral	602	16	–	**51**	67
	Negative	589	23	**54**	–	77
	Number of trials	1782				180
2°	Positive	2268	–	121	**154**	275
	Neutral	2274	92	–	**478**	570
	Negative	2250	253	**396**	–	649
	Number of trials	6792				1494
4°	Positive	2122	–	**303**	223	526
	Neutral	2096	327	–	**408**	735
	Negative	2126	519	**599**	–	1118
	Number of trials	6344				2379

For each condition the number of trials with incorrect (wrong) responses. In bold the most frequent type of error per condition. Analysis based on trials with fixation within 1° from center. Note that “positive valence” response was not the most frequent wrong response in any condition, ruling out the possibility of the results being driven by a “positive valence” response bias. Furthermore, in 3 of the 6 parafoveal conditions the majority of the errors (wrong responses) were of a “negative valence” response, ruling out the possibility that the results are a consequence of refraining from responding “negative valence” in the parafovea.

Lastly, we examined whether lower-level vision (visual acuity) or higher-level vision (face memory) abilities could potentially account for the results we found. Binocular visual acuity measures (block-design experiment (n = 49): mean VA acuity =  − 0.1 logMAR ± 0.15 (SD); single-trial experiment (n = 35): mean VA acuity =  −0.1 logMAR ± 0.10 (SD)) were not correlated with the face valence categorization accuracy at 0° eccentricity (p’s > 0.135). Similarly, accuracy in the Cambridge Face Memory Test (block-design experiment (n = 43): 72.09% ± 12.55% (SD), single-trial experiment (n = 31): 71.74% ± 12.15% (SD)) was not associated with face valence performance at the center (p’s > 0.22).

## Discussion

In this study we investigated the effect of eccentricity on valence judgements for emotional faces in the parafovea and found that eccentricity affects, as expected, valence judgements, but its effects are modulated by valence such that positive valence was least affected by eccentricity and negative valence the most. These results were consistent across 2 experimental paradigms and were not a result of speed-accuracy tradeoff (positive valence had higher accuracy and faster responses), of response biases towards positive or away from negative valence, or related to low-level visual acuity or high-level face memory performance. In addition, we also found that within-valence but not across-valence parafoveal performance was associated across different eccentricities (2° and 4°) indicating on dissociated mechanisms supporting perception of the different valence types.

The fact that performance reduced with eccentricity, even for face valence judgements, is not surprising. Earlier studies with low to high-level visual functions have also shown that visual performance decreases with growing eccentricity (e.g.^14,15,32,33^). In an earlier study we found that upright neutral face discrimination performance is reduced in the parafovea (4°) by ~ 10%^15^. Here we found that face valence performance in the parafovea (4°) was reduced to a greater extent with ~ 20% on average in the blocked-design experiment and ~ 27% on average in the single-trial experiment. It has been suggested that cortical magnification factor^43^ may account for reductions in performance in peripheral vision^32,33,44^. In line with the idea that cortical magnification contributes to peripheral performance reductions, earlier studies using emotional face stimuli bigger than ours in the periphery found accuracy levels higher than ours. For example, one study using bigger faces (~ 15°) found that peripheral performance for larger eccentricities (15°–30° to the right and left of fixation) decreased with eccentricity but was overall much higher than in our study (87–95%)^26^. Another study investigating how peripheral fearful faces are processed and perceived at similar eccentricities (15°–30° to the right and left of fixation) using enlarged emotional face stimuli (width > 15°) found reduction in performance with eccentricity^27^ with much higher accuracies than in our study. Another study using big emotional face stimuli (~ 7.5°) also found decreased peripheral performance with 80% accuracy at 10° eccentricity^34^, higher than the accuracy levels we find at 4°. Since in everyday life faces retain their world size across the visual field and thus are not enlarged in peripheral vision, here we adopted this naturalistic approach of investigating how eccentricity, without compensating for cortical magnification, affects valence performance^45^. The face stimuli we used subtended 2° × 2.71° corresponding to the size of a real face when viewed from a distance of ~ 4 m^45,46^. The performance reductions found in our study are unlikely to be solely explained by the cortical magnification factor given the profound differences in eccentricity-based reductions found here relative to those found for face discrimination in the same parafoveal locations^15^ and given our current results where performance was modulated by eccentricity according to valence. Recent studies relate to the possibility that emotional information may influence sensory and attention-related processes^5^ and some even suggest that this may relate to action related processes when information is task-relevant^9–12^. In our study, we found that perceptual accuracy for each emotional valence was modulated differently by eccentricity while response times of each valence were affected by eccentricity in a similar manner. Since in our study emotional information was only task relevant and we did not directly modulate attention, it is hard to predict whether influences of emotional information on sensory and attention-related processes would generalize to the periphery and whether they would be differentially modulated by valence or emotional content.

While there was an overall reduction of performance with eccentricity, different valence categories were found to be affected in a different manner by eccentricity across the different experimental designs we used, suggesting that different valence categories are supported by different mechanisms. While the average reduction was greater than that reported for face discrimination (see above), when we examined performance by specific valences we found that positive valence was affected the least by eccentricity (with an average reduction of ~ 10% at 4° in the block-design experiment and ~ 19% in the single-trial experiment), and negative valence the most (with an average reduction of ~ 35% at 4° in the block-design and ~ 38% in the single-trial experiment). While potentially speed-accuracy trade-off differences could account for the differences in performance reduction by valence type, the reaction times analyses suggest that this is not the case here, as positive valence was both most accurate and had fastest reaction times, while negative valence had worst accuracy and slowest reaction times in the parafovea. A response bias analysis examined the possibility that the different eccentricity-modulations in performance were due to response bias favoring positive expression responses over negative ones and found that this was not the case. A further correlational analysis we ran revealed that parafoveal performance was correlated across eccentricities within valence type, but not across valence types. While the received view is that emotional stimuli are of vital importance and are thus processed differently than neutral stimuli, it is still unclear what factors underlie heightened performance for one valence over another. It has been suggested that emotions stimulate two different motivational systems, one appetitive that is associated with positive emotional stimuli and another defensive that is associated with negative emotional stimuli^6,7,47^. Such models may even lead to the assumption that negative emotional stimuli may be more important for survival and therefore may result in higher performance for negative emotional stimuli even in parafoveal vision, similarly to what has been found for central vision in some studies^48–50^. However, not all studies support this assumption. For example, there are studies that suggest that positive emotions lead to higher performance for central and parafoveal stimuli, and these are in line with our findings. One earlier study with a different paradigm and stimuli^25^ examining only one peripheral eccentricity (2.5°) on the horizontal meridian (i.e. right or left of fixation) reports that performance for happy faces is fastest and most accurate, consistent with our findings. Another study investigating only negative and neutral faces at much greater eccentricity than ours (at 15°–30° to the right or left of fixation) reports on eccentricity modulation of performance and that negative expressions were recognized less accurately than neutral ones^27^, in line with our results. Additional studies investigating face expressions in foveal vision also report on superior performance for positive stimuli (e.g. Ref.^51^). However, another group of studies investigating different effects of emotional information presented centrally do not find differences between the effects of positive and negative valence. One recent study suggests that observation of emotional faces with positive or negative valence may prime the body for action as evident by enhancement of corticospinal excitability^52^, but no differences between positive and negative valence faces were found. Another study investigated similar effects in response to emotional body postures and again found a significant difference in motor evoked potentials between emotional bodies and neutral bodies but no difference between positive and negative valence^53^. Other studies also suggest that emotional stimuli, presented either directly or indirectly, can influence multiple processes related to action, motor control or bodily responses^54–56^. Since most of these studies used central (rather than peripherally presented) stimuli, given our findings of valence-dependent modulations mostly evident in peripheral vision, it could be interesting to examine if such motor-related modulations are affected by eccentricity in a similar manner to perceptual processes. Such findings may have implications for motor and action related behaviors of older adults with constricted peripheral vision^57,58^ that are also prone to falls^59,60^. It is hard to predict if our results that are based on shortly presented stimuli may generalize to longer presentation durations that better mimic naturalistic conditions. Nevertheless, our paradigm and different analyses suggest that investigations of parafoveal vision may be essential to reveal differences not evident at central vision, and that the different valence categories examined here with brief presentation durations are supported by dissociated mechanisms.

### Limitations and future directions

While some factors (mentioned above) are unlikely to explain our results, we cannot rule out the possibility that the results we found are expression-specific rather than valence-specific. In this study we used three different expressions, each representing unique expression and valence. Additional within-valence expression types shall be examined to determine whether the effects reported here are indeed valence- or emotion-specific. Nevertheless, the results do suggest that differences across valences are likely to exist, given the differences we found here between three valence-representative emotions. We cannot rule out the possibility that the differences between the positive and negative valences we have found are due to arousal aspects that have been suggested to influence emotional recognition and response related processes^61^, to differences between the positive and negative stimuli as their level of valence, arousal, or emotional recognizability, or to the dataset the images were taken from. Additional research is needed to address these and additional potential confounds in order to examine whether the results we report here replicate when potential confounding factors are controlled for.

## Conclusions

Our results suggest that investigations of peripheral vision can expose processing differences that may not be evident at foveal vision due to ceiling effects. While parafoveal vision can expose associations and dissociations between different visual tasks (as evident here and in Ref.^15^), further peripheral eccentricities may lead to floor performance effects and thus it is unclear at this point how informative further peripheral investigations (beyond the parafovea) may be. Further research is required to substantiate the results reported here to assess their relevance to behaviors of older adults with constricted peripheral vision that are also prone to falls.

## Supplementary Information

Supplementary Information.

## Data Availability

The datasets generated and/or analysed during the current study are available in the Open Science Framework repository at https://osf.io/8t6r2/.
