# Supplementary material for: Positive and negative facial valence perception are modulated differently by eccentricity in the parafovea
Source: Sci Rep. 2022 Dec 15;12:21693. doi: 10.1038/s41598-022-24919-7 (PMC9755278; doi:10.1038/s41598-022-24919-7)
Supplement: Supplementary file 1 — Supplementary Information. [file 41598_2022_24919_MOESM1_ESM.pdf]

Supplementary Material

Positive and negative facial valence perception are modulated differently by eccentricity in the parafovea

Vasilisa Akselevich[1,2], Sharon Gilaie-Dotan[1,2,3,*]

[1] *School of Optometry and Vision Science, Bar Ilan University, Ramat Gan, Israel*

[2] *The Gonda Multidisciplinary Brain Research Center, Bar Ilan University, Ramat Gan, Israel*

[3] *UCL Institute of Cognitive Neuroscience, London, UK*

Supplementary Results

Block design analysis: Comparison between analyses with and without outliers

A two-way repeated measures ANOVA was run to determine the effects of eccentricity and emotional valence on emotional expression categorization accuracy and response time. In the analysis of accuracy there were seven outliers, which had a studentized residual value of more than ± 3. After comparing the results of two-way repeated measures ANOVA with and without the outliers included in the analysis, we found that both results did not differ sufficiently for different conclusions to be drawn from the data.

Eccentricity and valence effects on accuracy analysis with these outliers (as reported in the main Results)

Eccentricity: $F(2, 100) = 94.9$, $\boldsymbol{p < .001}$, $\eta^2 = .655$

Valence: $F(2, 100) = 18.29$, $\boldsymbol{p < .001}$, $\eta^2 = .268$

Interaction: $F(3.032, 151.601) = 16.187$, $\boldsymbol{p < .001}$, $\varepsilon = .758$, $\eta^2 = .245$

Corresponding accuracy analysis without outliers

Eccentricity: $F(1.7, 73.3) = 97.65$, $\boldsymbol{p < .001}$, $\varepsilon = .852$, $\eta^2 = .694$

Valence: $F(2, 86) = 15.562$, $\boldsymbol{p < .001}$, $\eta^2 = .266$

Interaction: $F(2.627, 122.977) = 15.836$, $\boldsymbol{p < .001}$, $\varepsilon = .657$, $\eta^2 = .269$

Eccentricity and valence effects on RT analysis with these outliers (as reported in the main Results)

Eccentricity: $F(1.642, 82.125) = 10.286$, $\boldsymbol{p < .0001}$, $\varepsilon = .821$, $\eta^2 = .171$

Valence: $F(1.777, 88.862) = 2.845$, $p = .07$, $\varepsilon = .889$, $\eta^2 = .054$

Interaction: $F(3.237, 161.843) = 1.101$, $p =.353$, $\varepsilon = .809$, $\eta^2 = .022$)

Corresponding RT analysis without these outliers

Eccentricity: $F(1.581, 67.964) = 9.605$, $\boldsymbol{p = .001}$, $\varepsilon = .79$, $\eta 2 = .183$

Valence: $F(2, 86) = 1.521$, $p = .224$, $\eta^2 = .034$

Interaction: $F(2.955, 127.047) = 1.041$, $p = .376$, $\varepsilon = .739$, $\eta^2 = .024$

Single-trial analysis: Comparison between analyses with and without outliers

In the accuracy analysis, there were two outliers, which had a studentized residual value of more than ±3. After comparing the accuracy results of two-way repeated measures ANOVA with and without the outliers we found that both results did not differ sufficiently for different conclusions to be drawn from the data.

Eccentricity and valence effects on accuracy analysis with the 2 outliers (as reported in the main Results)

Eccentricity: $F(1.453, 52.298) = 208.7$, $\boldsymbol{p < .001}$, $\varepsilon = .726$, $\eta^2 = .853$

Valence: $F(2, 72) = 19.795$, $\boldsymbol{p < .001}$, $\eta^2 = .355$

Interaction: $F(2.745, 98.824) = 9.082$, $\boldsymbol{p < .001}$, $\varepsilon = .686$, $\eta^2 = .201$

Accuracy analysis without the 2 outliers

Eccentricity: $F(1.411, 47.966) = 200.594$, $\boldsymbol{p < .0001}$, $\varepsilon = .705$, $\eta^2 = .855$

Valence: $F(2, 68) = 22.091$, $\boldsymbol{p < .0001}$, $\eta^2 = .394$

Interaction: $F(2.783, 94.638) = 10.426$, $\boldsymbol{p < .0001}$, $\varepsilon = .696$, $\eta^2 = .235$,

Corresponding RT analysis with the 2 outliers (as reported in the main Results)

Eccentricity: $F(1.415, 50.95) = 13.666$, $\boldsymbol{p < .001}$, $\varepsilon = .708$, $\eta^2 = .275$

Valence: $F(2, 72) = 21.519$, $\boldsymbol{p < .001}$, $\eta^2 = .374$

Interaction: $F(2.587, 93.119) = 0.505$, $p = .652$, $\varepsilon = .647$, $\eta^2 = .014$

Corresponding RT analysis without the 2 outliers

Eccentricity: $F(1.387, 47.141) = 17.612$, $\boldsymbol{p < .001}$, $\varepsilon = .693$, $\eta^2 = .341$

Valence: $F(2, 68) = 19.429$, $\boldsymbol{p < .001}$, $\eta^2 = .364$

Interaction: $F(2.736, 93.038) = 0.422$, $p =.72$, $\varepsilon = .684$, $\eta^2 = .012$
